# Neuropsychological profile in acromegaly: a single center cross sectional analysis and preliminary prospective long-term study

**DOI:** 10.3389/fneur.2026.1736577

**Published:** 2026-03-03

**Authors:** Giorgia Abete Fornara, Alessandra Mangone, Veronica Lotito, Giulia Del Sindaco, Arianna Cremaschi, Giulia Carosi, Roberta Mungari, Emanuele Ferrante, Giulio Andrea Bertani, Giorgio Fiore, Marco Locatelli, Elisa Sala, Giovanna Mantovani

**Affiliations:** 1 Unit of Neurosurgery, Fondazione IRCCS Ca' Granda Ospedale Maggiore Policlinico Milan, Milan, Italy; 2 Endocrinology Unit, Fondazione IRCCS Ca’ Granda Ospedale Maggiore Policlinico Milan, Milan, Italy; 3 Department of Medical Sciences and Community Health, University of Milan, Milan, Italy; 4 Department of Pathophysiology and Transplantation, University of Milan, Milan, Italy

**Keywords:** acromegaly, cognitive function, hypopituitarism, neuropsychology, pituitary

## Abstract

**Background:**

Psychological and cognitive disorders have been reported in acromegaly, yet with limited and heterogeneous data, especially concerning long-term cognitive functioning.

**Methods:**

We conducted a cross-sectional study enrolling 44 acromegalic patients and 40 healthy controls. We systematically assessed anxiety and depressive symptoms through the State–trait Anxiety Inventory and the Beck Depression Inventory, respectively. We investigated their cognitive functioning thorough a wide battery of 16 tests addressing verbal and visuo-spatial memory, attention, verbal fluencies, executive functions and constructional praxis. Moreover, we performed a prospective evaluation in a 10-year time-span of a small subgroup of patients.

**Results:**

Clinically significant depressive and anxiety symptoms were registered in 23 and 35% of patients respectively, mostly in the group with active disease at evaluation. Concerning cognition, patients scored worse than controls in all cognitive domains explored, with a significant difference registered in almost all tests administered. Moreover, hypopituitarism and IGF-1 levels seem to be related to a worse cognitive performance, especially in the group of tests exploring the memory domain. In the prospective group, with the limitation of a really small sample size, we observed a global improvement over time in all domains evaluated.

**Conclusion:**

Acromegaly is characterized by higher levels of psychological distress and poorer neurocognitive functioning, with a possible association with activity of disease.

## 1 Introduction

Acromegaly is a rare disease resulting from chronic exposure to high levels of growth hormone (GH) and its mediator insulin-like growth factor 1 (IGF1), caused in almost all cases by a GH-secreting pituitary tumor (1). GH and IGF1 excesses induce several body changes and a wide range of systemic effects, such as cardiovascular, pulmonary or metabolic comorbidities, resulting in increased morbidity and mortality and reduced quality of life (1–3).

Psychopathological distress is emerging in literature as a significant burden among acromegalic patients, mostly in terms of depression or anxiety disorders, even though subtle to detect (4–7). Therefore, an assessment of the psychological status has recently been suggested to be systemically carried out in affected patients (7, 8). The local effects of the tumor, the hormonal changes and the specific therapies have all been accounted for the pathogenesis of these findings, yet specific cause-effect relations remain to be proven (9).

Moreover, impairment in cognitive functions is often reported in acromegaly, as already described since the early 1990s (10). In particular, different studies reported alterations in memory and executive functions, attentive abilities and information processing speed as the main impairments (8, 10–12).

These findings have been correlated to disease duration, patient’s age and gender and to structural brain abnormalities in medial temporal lobe (4, 8, 13). However, results are highly heterogeneous and scarcely comparable, as underlined by a recent review by Pertichetti and colleagues, due to important differences in terms of population, extent of neuropsychological assessments and tests administered among the different data available in literature (14).

The aim of the present study is to deeply assess the neuropsychological functioning and mental well-being of patients affected by acromegaly, to provide a comprehensive and detailed cognitive assessment through an extensive neuropsychological battery composed by well-established tests, whereas in literature previous studies addressed only few cognitive domains with often simple and unspecific tests. We also provide a direct comparison with a healthy-subject matched group, in order to describe qualitative differences in terms of cognitive functioning, to focus also on subtle neuropsychological differences and not only on the quantitative scores, as generally provided previously. Additionally, we prospectively studied a small subgroup of patients for 10 years, in order to provide a first preliminary description of their cognitive functioning over a long-term period.

## 2 Materials and methods

### 2.1 Study cohort

We conducted a cross-sectional study recruiting 44 acromegalic adult patients referring to a single tertiary center. Patients were recruited between 2011 and 2023 and were diagnosed with acromegaly according to international guidelines (1, 15).

Thirty-one patients of our cohort were also included in a previous multicentric study, yet the present study includes 9 additional neuropsychological tests, exploring 5 additional cognitive domains. In particular, the following test have been added: Rey complex figure test, Rey Auditory verbal Learning test, Stroop test, Wisconsin Card Sorting Test, Weigl test, Semantic Fluencies, Naming of Object test, Token test, Attentional Matrices. Moreover, the inclusion of a healthy control group whose cognitive performance is directly compared to that of the patients’ group, enables us to study cognition not only from a quantitative approach, but also from a qualitative perspective.

We included acromegalic patients of age ≥18 years willing to perform a complete psychological and cognitive evaluation at recruitment. Exclusion criteria were: (i) a prior history of neurological or psychiatric diseases, (ii) assumption of psychiatric drugs, alcohol and/or drug abuse, (iii) severe visual or auditory impairments.

Moreover, we performed a prospective analysis of a small subgroup of 9 patients enrolled at diagnosis and followed-up, 6 of them up to 10 years. All patients underwent transsphenoidal pituitary adenoma surgery (TNS), which was performed at the Neurosurgery Unit of our Center. They underwent a complete psychological and cognitive assessment at four different time-points: (T1) at diagnosis, before any active intervention; (T2) 3–4 days post-operatively; (T3) 12-months after surgery; (T4) 10 years after surgery. In these cases, parallel versions of the tests were used when available, to avoid learning effect. A cohort of 40 adult healthy control subjects was also recruited, comparable for sex, age, and educational level. Controls were assessed with the same neuropsychological battery of patients. The previous presence of any medical or psychiatric condition which may influence the cognitive functioning was excluded by performing a complete psychological and medical anamnesis prior to the evaluation.

A written informed consent was signed by each participant. The local ethic committee approved the study (Comitato Etico Milano Area 2, approval number 973_2019bis, 10/10/2019).

### 2.2 Study protocol

Acromegalic patients completed the following self-report questionnaires investigating anxiety and depression:

- Beck Depression Inventory-II (BDI), a widely used instrument to investigate depressive symptoms; a score ≥ 14 indicates the presence of clinically significant depressive symptoms (16);
- State–Trait Anxiety Inventory forms X1 and X2 (STAI X1 and X2) to address state and trait anxiety levels, respectively (17).

In STAI X1 a score equal or superior to 65 or 71 was considered pathological for males and females, respectively. In the STAI X2 a cut-off of 56 and 62 represented pathological anxiety for males and females, respectively.

Both patients and controls completed an extended battery of 12 neuropsychological tests, addressing 14 cognitive functions, standardized for the Italian population, administered and corrected by a single dedicated neuropsychologist.

We tested short- and long-term verbal and visuo-spatial memory, selective attention and attentional shifting, verbal fluencies, inhibitory functions, constructional praxis, deductive reasoning and perseverative behavior. The complete test list with normality scores is detailed in Table 1. To avoid learning effect, parallel forms were used when available for the clinical practice.

**Table 1:** Complete list of the neuropsychological tests administered, divided into cognitive domains.

Cognitive domain	Test	Function tested	Normal cut-off values
Memory	Digit Span forward (34)	Short-term verbal memory	>4.26
	Corsi span (34)	Short-term visuo-spatial memory	> 3.46
	Rey complex figure-recall (35)	Long-term visuo-spatial memory	>9.46
	Rey 15- word list (RAVLT) immediate Long-term recall (36)	Auditory learning Long-term verbal memory	>28.51 >4.67
Executive and frontal functions	Digit Span backward (34)	Verbal working memory	>2.65
	Stroop Test (37) Time score Errors score	Cognitive inhibition and flexibility	< 36.92 <4.24
	Wisconsin Card Sorting Test (WCST) (38) Global score Perseverative errors	Deductive reasoning and perseverative behavior	< 90.6 < 42.7
	Weigl Test (18)*	Abstraction and classification	≤ 8.0
	Semantic Fluencies (39)	Verbal fluency trough semantic cue	>23.58
Language	Phonemic Fluencies (40)	Verbal fluency trough phonemic cue	> 16
	Catricalà Naming of Object (19)*	Word production	≤ 41.48
	Token Test (20)*	Oral comprehension	≤ 26.25
Attention	Trail-Making Test (TMT) (41) Sub-test A Sub-test B Sub-test B-A	Visuo-spatial selective attention and attentional shifting	< 93 < 282 < 186
	Attentive Matrices (18)*	Visuo-spacial research and selective attention	< 30
Praxis	Rey complex figure-copy (36)	Constructional Praxis	> 28.87

^*^Indicates tests administered only to the longitudinal subgroup.

The neuropsychological and psychological assessment took approximately 1 h; raw scores of the cognitive tests were corrected for age, educational level and gender when appropriate, according to Italian normative data, obtaining the adjusted scores (AS) used in the statistical analysis; equivalent scores (ES) were also calculated for qualitative analyses, as they enable to easily detect whether the performance is normal (ES = 2,3,4), borderline (ES = 1) or pathological (ES = 0), representing a ordinal scale. AS indicate the score obtained for each test and are represented by a continuous score.

AS, due to their test-specific scaling and psychometric properties, are difficult to compare directly across instruments and domains. In contrast, the use of a categorical parameter such as ES allowed us to schematize results across an extensive neuropsychological battery within a common metric, facilitating domain-level synthesis. For this reason, ES were adopted as the primary framework for cross-test and domain-level interpretation.

The cognitive evaluation administered to the prospective subgroup consisted in 4 additional tests investigating abstract reasoning: Weigl test (18), object-naming [Catricalà naming test (19)], verbal comprehension [Token test (20)] and visuo-spatial attention [Attentive Matrice (18)]. Such a wide battery was chosen in order to widely study the neuropsychological profile of patients and to overcome the limit of short assessments often reported in literature.

### 2.3 Clinical data

Clinical, demographical, neuroradiological and biochemical data were retrospectively extracted from medical records. IGF1 values were compared with an appropriate age- and sex-adjusted range and expressed as percentage of the upper limit of normal (ULN) for individual laboratory samples.

Acromegaly was considered in remission in the presence of normal IGF1 levels, measured at least 6 weeks postoperatively according to current guidelines, and GH values after OGTT <0.4 μg/L (1, 21, 22).

Disease status was recorded at diagnosis, at T3 (12 months after surgery) and T4 (10 years after surgery). Disease at T2 was not assessed as clinically and biochemically uninterpretable as to close to surgery as suggested form acromegaly guidelines (1).

Patients were considered controlled if during therapy mean fasting GH levels were below 1 μg/L in the presence of normal IGF1 levels (IGF1 < 1.2 × ULN), whereas in the remaining cases they were classified as uncontrolled (1, 22). IGF1 levels only were considered in patients receiving pegvisomant.

Patients were divided into three groups according to disease status at psychological evaluation as follows: active disease, comprising both naïve and uncontrolled patients, disease remission and controlled disease. As the main clinical outcome in acromegaly is the normalization of IGF-1 levels, for statistical purposes patients were grouped into two main categories: patients with elevated IGF-1 levels were considered as having active disease (including naïve patients and those uncontrolled during therapy), while patients with normal IGF-1 levels were considered as having controlled/cured disease (including both patients in disease remission and those controlled during therapy).

Hypopituitarism was defined as the presence of one or more hormonal deficit. Hypoadrenalism was defined by an abnormal response of cortisol in a dynamic test. In particular, before June 2016 we considered inadequate a peak of cortisol 500 nmol/L, during 1 μg corticotrophin stimulation test (ACTH 1 mcg) or insulin tolerance test (ITT); while after that time point the cut off according to the kit used in out Institution (Roche 2) changed to 351 nmol/L (23–25). A free thyroxine (fT4) under reference range in combination with not adequately increased TSH concentration was indicative of TSH deficiency (26, 27). Central hypogonadism was suggested in premenopausal women with low estradiol and low LH/FSH in conjunction with oligomenorrhea or amenorrhea, and in postmenopausal women in the presence of FSH levels inappropriately low for menopausal status. In men, central hypogonadism was defined by testosterone under reference range for age and normal/low LH/FSH in combination with clinical signs and symptoms (26). All hormonal deficits were appropriately treated with hormonal replacement therapy.

### 2.4 Statistical analysis

Continuous parameters were described as mean±standard deviation (SD) whe normally distributed and as median and interquartile range (IQR) when non-normally distributed. Normality of data distribution was assessed using the Shapiro–Wilk test. Continuous data were compared using *t*-test or Wilcoxon–Mann–Whitney test, as appropriate. Categorical data were presented as percentage (%), proportion (/) and analyzed using the Chi-squared test or Fisher’s exact test as appropriate.

Linear Regression analysis was performed to analyze the association between a series of endocrinological data (hormonal deficits, IGF-1 levels, disease status) and neuropsychological status, using the AS for quantitative analysis and the ES for qualitative descriptions. Friedman tests were conducted on longitudinal subgroup to examine the modification of cognitive function over time.

When Friedman test showed a significant overall effect, post-hoc Wilcoxon tests with Bonferroni’s correction were performed to account for multiple comparisons between different time-points.

*p*-values < 0.05 were considered statistically significant. Statistical analyses were conducted using GraphPad Prism 7.0 (GraphPad Software) and IBM SPSS 28.0 (IBM Analytics).

## 3 Results

### 3.1 Cross-sectional analysis

Demographical and clinical data of the patient’s cohort are detailed in Table 2.

**Table 2:** Clinical and sociodemographic data of the patient cohort at the time of the cognitive evaluation.

Variable	Results
Age	57.30 ± 13.71 (28–79)
Education years	12.23 ± 3.92 (5–21)
Gender	*M* = 18 (41) *F* = 26 (59)
Neuroimaging	Microadenoma: 13 (29)
	Macroadenoma: 31 (70)
Presumed duration of acromegaly	7.7 ± 5.5 (1–30)
Therapies	None: 2 (4)
	TNS: 36 (81)
	Radiosurgery: 8 (18)
	Medical therapy: 22 (50)
IGF-1 (ULN)	1.34 (0.25–5.09)
Hormonal deficits	No deficit: 29 (66)
	Hypopituitarism: 15 (34)
Clinical status	Not controlled/active: 13 (29.)
	Controlled: 19 (43)
	In remission: 12 (27)

All data are expressed as mean ± SD (range) or number (%).

M, male; F, female; TNS, transphenoidal surgery.

IGF1 are expressed as upper limit of normal (ULN).

The control group was composed by 40 healthy subjects (24F/16M), with a mean age of 52.43 ± 12.06 years and a mean educational level of 12.77 ± 3.11 years. These variables did not differ between patients and controls (*p* = 1.0, *p* = 0.089 and *p* = 0.494, respectively).

### 3.2 Psychological status

In the group of acromegalic patients, mean BDI score was not indicative of significant depressive symptoms (9.07 ± 8.08, range 0–39) considering its normative data. However, 10 patients (22.7%) reported a pathological score: in particular, 8 patients (18%) scored as mild and 2 patients (4%) as severe symptomatology. Fifty percent of patients scoring pathologically were in the active disease group (*p* = 0.13 vs. controlled/cured).

Analyzing results of anxiety questionnaires, the mean STAIX1 score in males was 53.17 ± 16.59, and in females was 50.42 ± 9.9, indicating a globally not significant state anxiety.

**Figure 1:**
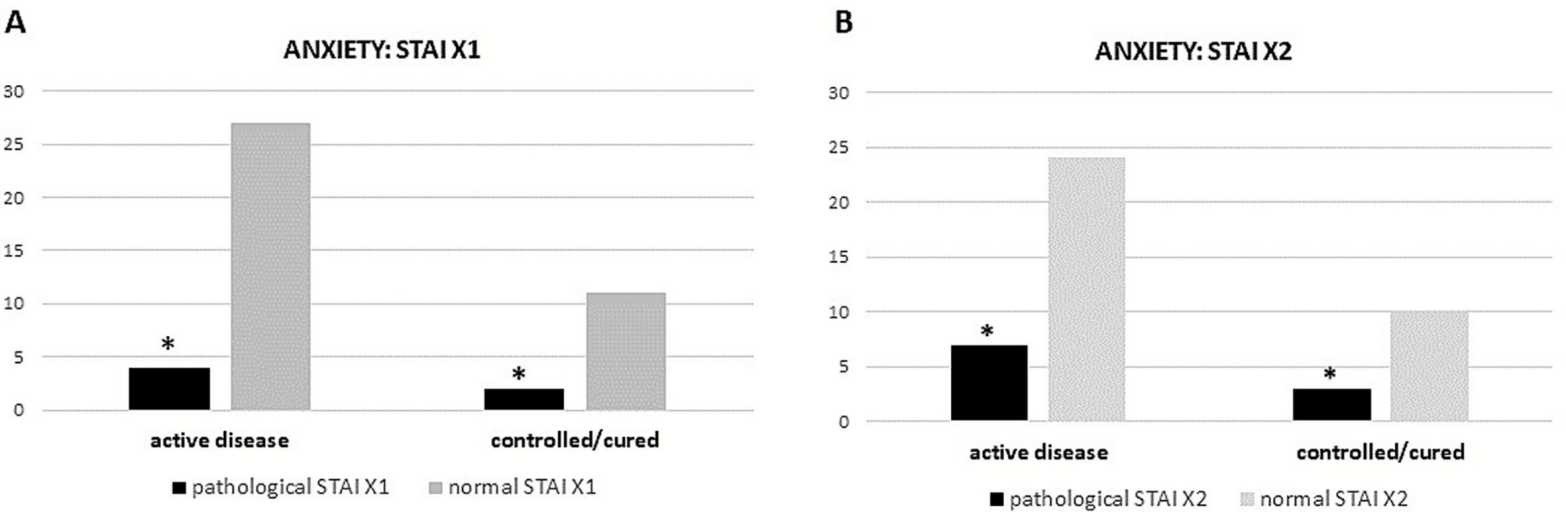
Results of STAIX1 **(A)** and 2 **(B)** in acromegalic patients divided according to disease status at evaluation. *p < 0.05.

However, 6 males (33.5%) scored pathologically at STAIX1 test, while no female reported a clinical state of anxiety (STAIX1 female mean 50.42 ± 9.9). Four out of 6 patients (67%) with pathological scores were in the active disease group (*p* = 0.05 vs. controlled/cured), as shown in Figure 1A. On the contrary, mean results of STAIX2 indicated a slight general trait anxiety (52.22 ± 17.96), with 7 patients (38.9%) reporting a pathological score, 6 (85%) of whom were females. Even analyzing the STAIX2 results, the majority of patients with pathological scores were in the active group (70%) with a significant difference compared to the controlled/cured group (*p* = 0.017) (Figure 1B).

The linear regression analyses showed a significant correlation between IGF-1 levels and STAIX1 and X2 scores (*p* = 0.01, *B* = 0.64 and *p* = 0.008, *B* = 0.74 respectively), indicating that higher levels of the hormone are associated to higher levels of anxiety. On the contrary, no relation was found between the BDI test and IGF1 levels.

Interestingly, we also found a significant negative correlation between age and STAIX1 (*p* = 0.046, *B* = −0.29) and STAIX2 scores (*p* = 0.042, *B* = −0.33), indicating that younger acromegalic patients tend to experience more anxiety. No other clinical or demographical variables resulted significantly associated with depression or anxiety.

### 3.3 Neuropsychological assessment

Results of the neuropsychological tests are reported in Table 3.

**Table 3:** Results of the neuropsychologic tests in the two cohort (patients and controls).

Adjusted scores					Equivalent scores					
Test	Normal cut-off values	Patients mean score	Controls mean score	*p* ^*^	Patients PT/NO *N* = 44	Controls PT/NO *N* = 40	*p* ^§^	Patients PT+ BD/NO *N* = 44	Controls PT+ BD/NO *N* = 40	*p* ^§^
Corsi Test	> 3.46	4.44 ± 0.90	5.29 ± 0.90	**<0.001**	2/42	0/40	0.49	24/20	6/34	**<0.01**
Digit Span forward	> 4.26	5.38 ± 1.23	5.99 ± 0.96	**0.014**	9/35	1/39	**0.01**	12/32	6/34	0.46
Digit Span backward	> 2.65	3.85 ± 1.12	4.90 ± 0.76	**<0.001**	5/39	0/40	**0.05**	14/30	0/40	**<0.01**
Recall Rey figure	> 9.46	12.09 ± 5.44	15.93 ± 5.29	**0.002**	11/33	4/36	0.09	14/30	7/33	0.2
RAVLT-immediate recall	> 28.51	34.44 ± 9.10	49.84 ± 10.3	**<0.001**	12/32	1/39	**0.01**	21/23	2/38	**<0.01**
RAVLT-long-term recall	> 4.67	7.12 ± 2.15	11.26 ± 2.37	**<0.001**	6/38	0/40	**0.02**	13/31	1/39	**<0.01**
Semantic fluency	> 23.58	41.76 ± 9.25	50.19 ± 6.12	**<0.001**	1/43	0/40	1.0	4/40	0/40	0.11
TMT A	< 93	33.43 ± 22.18	25.05 ± 15.0	**0.046**	1/43	0/40	1.0	3/41	1/39	0.61
TMT B	< 282	143.4 ± 108.5	61.4 ± 51.43	**<0.001**	5/39	1/39	0.2	13/31	1/39	**<0.01**
TMT B-A	< 186	110.6 ± 103.2	36.9 ± 39.94	**<0.001**	9/35	1/39	**<0.01**	16/28	1/39	**<0.01**
Copy Rey figure	> 28.87	31.77 ± 4.74	32.82 ± 3.9	0.27	2/42	1/39	1.0	6/38	4/36	0.74
Phonemic fluency	> 16	28.48 ± 9.18	40.72 ± 9.48	**<0.001**	5/39	0/40	**0.05**	9/35	1/39	**0.01**
Stroop-time	< 36.92	29.19 ± 15.16	16.29 ± 7.80	**<0.001**	13/31	2/38	**<0.01**	15/29	2/38	**<0.01**
Stroop-errors	< 4.24	1.70 ± 1.73	0.36 ± 1.18	**<0.001**	4/40	1/39	0.36	8/36	1/39	**0.03**
WCST-global score	< 90.6	84.72 ± 39.87	29.74 ± 23.6	**<0.001**	24/20	0/40	**<0.01**	26/18	1/39	**<0.01**
WCST-perseverative errors	< 42.7	31.40 ± 19.09	9.80 ± 7.93	**<0.001**	12/32	0/40	**<0.01**	19/25	0/40	**<0.01**

Data are expressed as mean score ± standard deviations for the adjusted scores and as cognitive performance of patients vs controls in terms of equivalent scores.

*N*, number; BD, borderline performance (equivalent score = 1); PT, pathological performance (equivalent score = 0); NO, normal performance (equivalent score = 2–4). Bold represents significant *p* values.

^*^Obtained by *t*-test; ^§^obtained by X^2^ test.

Overall, mean results of both patients and controls resulted within the respective ranges of normality in all the tests administered, considering AS; nevertheless, observing ES of the two groups, the number of patients scoring as pathological or borderline was significantly higher compared to controls in almost all tests, as shown in Table 3. This finding indicates that, besides obtaining a normal AS, patients perform poorer than healthy subjects in all the test considered, except for the Constructional Apraxia test.

Considering clinical data, hypopituitarism seems to impact the memory domain. In particular, observing ES, in the RAVLT-Immediate test 8 out of 15 (53%) patients with hypopituitarism scored as pathological versus only 4 patients out of 29 (14%) in the group without deficit (*p* = 0.010) and in the RAVLT-long term 5/15 patients (33%) with hypopituitarism scored pathological versus 1/29 patient (3%) without deficit (*p* = 0.01). In the Rey complex figure-recall test, we registered a slight difference in pathological results (ES) between hipopituitaric patients (scoring worse) and those without deficits (*p* = 0.05), with a stronger significance when including borderline results (*p* < 0.01).

Even if no differences were found between disease activity status and neurocognitive performances, IGF-1 levels still positively correlated with the results of Corsi test (AS) (*p* = 0.014, *B* = 0.04), suggesting an influence of hormonal values on the short-term visual memory functioning.

### 3.4 Prospective analysis

The prospective subgroup was composed by 9 patients: 4 (44.5%) males and 5 (55.5%) females, with a median age at diagnosis of 47 years (range 32–65) and a median educational level of 13 years (range 6–17). Not all patients completed the follow-up period, with 2 patients lost at T3 and 1 patient lost at T4 evaluation. At T3, 2/7 patients (28.5%) were in remission of disease and 2/7 (28.5%) were controlled with medical therapy.

At T4, 2/6 patients (33.3%) were in remission of disease and 4 (66.77%) were controlled with medical therapy.

Overall, patients scored within the respective normal ranges in most tests at baseline, and improved their neurocognitive performance over the follow-up period.

In particular, *post hoc* analysis with Wilcoxon signed-rank tests showed a significant improvement between T1 and T3 evaluations in the following tests: Rey-Osterrieth Complex Figure Test Recall [9.25 (1.25–18.6) vs. 21 (10.2–23.25), *p* = 0.01], RAVLT-Immediate recall [38.3 (27.2–44) vs. 53.7 (39.2–58.8), *p* = 0.03], RAVLT-long-term recall [7.7 (5.5–9.1) vs. 12.2 (9.6–13), *p* = 0.01], Semantic Fluencies [38 (35–45) vs. 49 (46–58), *p* = 0.03], TMT A [34 (29–42) vs. 30 (20–35), *p* = 0.01], TMT *B* [102 (84–172) vs. 85 (37–95), *p* = 0.03], Phonemic Fluencies [32 (26–43) vs. 47 (32–61), *p* = 0.01], WCST-perseverative score [19.5 (11.3–54.7) vs. 11.3 (10.4–23), *p* = 0.03], prose memory [8.5 (6.5–10.85) vs. 19.5 (8.5–21.25), *p* = 0.03].

**Figure 2:**
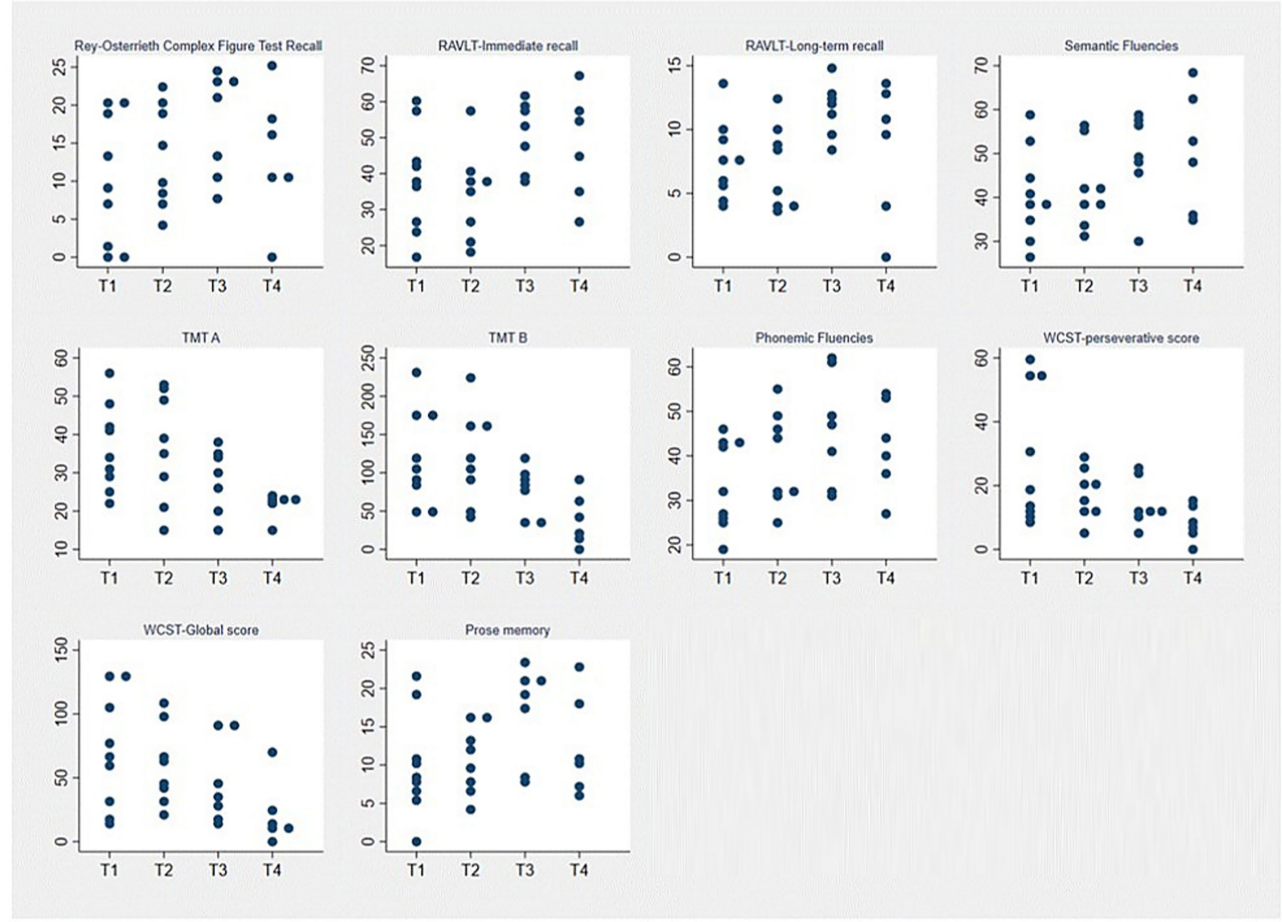
Neuropsychological evaluation in the total perspective group. Y-axis: Patients’ test scores. X-axis: Assessment time points (T1: at diagnosis; T2: 3–4 days post-operatively; T3: 12-months after surgery; T4: 10 years after surgery). Each dot corresponds to the performance of each patient.

Further significant effects emerged when comparing T1 and T4 for the TMT-A test [34 (29–42) vs. 23 (22–23), *p* = 0.01] and the WCST-Global score [66.5 (31.8–103.9) vs. 12.05 (11.2–23.4), *p* = 0.03]. As mentioned above, all these effects indicate a significant improvement over time, as shown in Figure 2.

Interestingly, in the Rey-Osterrieth Complex Figure Test Recall patients show a normal performance (mean 17.6 + 6.9) at T3 recovering from a borderline mean score at T1 (9.9 + 8.5).

No significant differences were found between T3 and T4 time points.

## 4 Discussion

Psychological and cognitive characteristics of acromegalic patients have been studied since the 1990s with heterogeneous results, mostly due to the small sample sizes and the use of limited batteries of tests administered (14, 28). However, despite the importance of neuropsychological symptoms, a specific evaluation in acromegalic patient struggles to enter into daily clinical practice and in the description of the symptomatic corollary of the disease, unlike what happens for other similar pituitary tumors such as Cushing’s disease. To our knowledge, this is the first study to provide such a deep and standardized investigation of cognitive functioning in acromegalic patients, based on an extensive neuropsychological battery of tests and a direct comparison to a healthy-subject group, revealing significant differences between patients and controls in the majority of the administered tests.

Concerning psychological dimensions, in our cohort 22% of patients showed elevated levels of depressive symptoms, a significantly higher percentage compared to the Italian population, where the prevalence of depression is around 5% (29). These findings are consistent with those reported in previous and recent studies (6–9). In particular, Cangiano *et al*. reported depressive symptoms in 28% of a large cohort of 171 acromegalic patients (8), while Pivonello et al. described a prevalence of approximately 30% in a multicenter cross-sectional study involving 223 patients (9).

Similarly, our data on anxiety are consistent with what previously described in the above-mentioned paper (8). We found a 33% prevalence of anxiety trait in acromegalic patients, in particular in the younger ones. This differs from the general population, where anxiety is more prevalent with aging, thus suggesting a possible direct role of the acromegalic disease in anxiety onset. Moreover, in our cohort anxiety symptoms were less evident in cured and controlled patients, with a significant correlation between IGF1 levels and anxiety scores. These findings further suggest a direct impact of disease on the psychological status, especially in young patients. Considering the self-report nature of the questionnaire administered, it is also possible that younger patients experience higher anxiety levels due to the initial reaction to the diagnosis and to those clinical pathways elderly patients are more confident with. On this matter, an interesting study confirmed the higher presence of psychological symptoms in a group of acromegalic patients compared to healthy subjects, and to patients suffering from other chronic non-endocrine pathologies, after exclusion of the impact of chronic diseases, therapies and medicalization on the psychological status (5).

It is interesting to notice that the important prevalence of psychological symptoms is comparable to what reported in literature and in a previous study conducted at our Institution on Cushing’s disease, a condition well known to be associated with psychological disorders (30–32), suggesting to take them in consideration also when addressing when addressing the disease burden of acromegaly.

Concerning the main aim of our study, patients scored within the normal range on quantitative cognitive measures. Nevertheless, when compared with healthy controls, they consistently showed poorer performance across nearly all administered tests. We can therefore speculate that cognitive functioning in acromegaly may qualitatively differ from that of healthy individuals, even in the absence of formal cognitive deficits.

In particular, poorer performances emerged in verbal memory, attention, executive functions and language production. Our extensive battery, together with the comparison with a control group, enabled us to qualitatively and quantitatively describe how all the cognitive domains assessed, except constructional praxis, were significantly different between the two groups, beyond what could be inferred from standardized normative data alone. The Italian multicenter study by Pivonello and colleagues obtained similar results on this issue (9), with a prevalence of deficits ranging from 9 to 13%, although their evaluation was limited to visuospatial and verbal working memory and verbal fluency. Despite the smaller sample size, our protocol includes nine additional cognitive tests, assessing further domains such as constructional praxis, long-term verbal and visual memory, inhibition, deductive reasoning and cognitive flexibility, abstractive reasoning, semantic fluencies, object naming and verbal comprehension.

One of the most interesting and new suggestion emerging from our analysis is the high percentage of borderline results in all neuropsychological tests in acromegaly, as showed in Table 3. This finding suggests the presence of subtle, subclinical cognitive impairment, which could not be detected without a specific and extensive assessment in a multidisciplinary Pituitary Unit, yet may impact significantly on patients’ daily life and treatment compliance.

Cognitive impairment in acromegaly appears to be more frequent with respect to patients with Cushing’s disease analyzed in a previous study by our group (32); this aspect could also possibly rely on the more widespread, albeit milder, impairment observed in acromegaly in the present work.

Unfortunately, it is not yet possible to suggest clear indications on pathogenetic causes of the global worsening of cognitive function in acromegaly, with some studies suggesting a role of hormonal factors, others to general and/or vascular problems such as presence of sleep apnea (5, 9, 33). Given the limited sample size, our findings can only indicate trends and suggestions, but it is interesting to note the possible contribution of active disease and hypopituitarism to poorer cognitive performance. In particular, our study evaluates for the first time the link between the presence of hypopituitarism and alterations in the specific field of memory, a coherent element if considering how temporal structures are sensible to hormonal changes in the cellular environment. The significant alteration of a specific neuropsychological domain in hipopituitaric patients may be related to specific hormonal alterations or activation of dedicated brain areas; however, this hypothesis remains speculative.

Overall, our study shows how mandatory a complete and wide neuropsychological assessment should be for acromegalic patients, as the subtle impairment we describe may not be detectable through general and non-specific investigations.

Considering the prospective sub-group, a general improving trend emerged in patients’ neuropsychological functioning, with a significant improvement in visuo-spatial long-term memory, verbal learning and long-term verbal memory, verbal fluencies, selective and alternate attention and deductive reasoning. Most of these tests are available in two or three different parallel versions, adopted in our protocol; thus, the improvement observed should not be directly linked with a learning effect; moreover, the long period of time between T2, T3, and T4 prevents the patients from this side effect. Above all, a quantitative improvement was registered for the long-term visuo-spatial memory, as patients generally reported a borderline score before surgery, while scored normally after 12 months. These normal results remained stable even after 10 years. Our data provide first important and reassuring insights into these patients’ long-term cognition, on which data are currently lacking, even if further research is needed with larger cohorts to generalize our first findings. Indeed, despite their clinical relevance, these findings should be interpreted cautiously due to the small sample size and the variable number of patients assessed at each time point.

In conclusion, our data, with the limitation of the small sample size enrolled, confirm a global alteration of both psychological and neuropsychological symptoms in the acromegalic population, in line with previous studies. Further studies with larger samples and extensive neuropsychological assessments are needed in order to better understand the underlined mechanisms and the long-term neuro-psychological function in acromegaly. Although preliminary and subject to the limitations discussed, our findings suggest that integrating psychological assessment into a dedicated multidisciplinary pituitary unit may contribute to a more comprehensive, patient-centered management of individuals with acromegaly.

### 4.1 Limitation of the study

We acknowledge that this study is limited by the retrospective nature of the analysis. The small number of subjects in this study limit the conclusions that can be drawn and preclude definitive affirmations.

## Data Availability

The raw data supporting the conclusions of this article will be made available by the authors, without undue reservation.
